# Observations on Life, as the Cause of the Vital Phenomena

**Published:** 1842-10

**Authors:** 


### ART. XVII.

*Observations on Life, as the Cause of the Vital Phenomena.—London,* 1842. 8vo, pp. 16.

THIS little pamphlet is intended, as we learn from its conclusion, but as the herald of a larger work, which, though nearly ready for publication, its author has been obliged to abandon for the present. The brochure has two claims on our attention: first, on account of the source whence it is understood to originate; second, as containing a new, and what will be considered by many a plausible, view of the nature of life, which professes to be founded upon experiments to be detailed. This view we shall present to our readers in the author's own language:

"He believes that the following inferences may now be considered as physiological facts:

"First, That there are a thousand things done in the body that cannot possibly be done by the mind, or by any other agent that resides in the brain. There must, therefore, be some other cause to produce these effects, otherwise the body could not be organized, neither could a single movement be made in the animal frame.

"Second, That there is in the living body, independently of the mind, an internal vital agent, which is endowed with an innate power of action; and that this vital agent, or in other words LIFE, is the direct cause of every movement, both voluntary and involuntary, that is made in the living animal.

"Third, That in man, and all the higher order of animals, the great solar ganglion is the seat of life; and the ganglionic nerves, which have their insertion in, and derive their origin from, this central organ, are the nervous machinery with which it works. The principle of action which resides in the solar ganglion has many motor nerves at its command, by which it performs its own voluntary movements; but the great sympathetic nerves, or the internal spinal cords, are the true motor tracts." (pp. 5-6.)

Now, in regard to the first of these propositions, we imagine that there can be no difference of opinion; since the old Stahlian doctrine, that *the soul* is concerned in all the operations of the organic system has been long since abandoned. But we need scarcely tell our readers that we utterly dissent from the second; and that consequently the third cannot in our opinion be true. We have on many occasions pointed out the absence of necessity for any such hypothesis as that offered by our author, in the explanation of the phenomena of the living organized body; and have shown that in the *microcosmus*, no less than in the *macrocosmus*, all sound philosophy would lead us to refer the phenomena which come under our notice, to *laws* dependent on the *properties* with which matter was originally endowed by its Creator. If in the one we require the hypothesis of a distinct entity or "organic agent,"—whether endowed, as Dr. Prout supposes, with faculties "little short of intelligence," or as our author more boldly states with "innate intelligence,"—we do in the other also. Intelligence implies consciousness. Does our author mean that this agent is conscious of its operations? In that case every animal must have within it two conscious entities and separate volitions—his mind, and his life; although his own consciousness only leads him to infer the presence of one. There seems to us to be no end to the absurd contradictions into which the assumption of any such doctrine must lead us. Our author attempts to get rid of these, however, by a kind of inversion of the Stahlian hypothesis; for, instead of considering the various operations of organic life as a sort of branch-office of the soul, he regards the mind itself as only one manifestation of his "vital agent."

"Life," he says, "has its faculties as well as the mind; and the mind is but one of the many faculties of the internal vital agent. The mind is only a part; life, or the vital entity, is the whole. It is the mind that enables the vital entity to keep up a connexion with the external world. It is the mind to which the vital agent is indebted for all its acquired knowledge. Selective absorption, digestion, circulation, assimilation, organization, respiration, secretion, reparation, and reproduction, are all vital properties. Sensibility is common to both the vital and mental powers; but even this is decidedly a vital property. Consciousness, memory, thought, reason, judgment, and volition, are mental powers, but these powers are merely superadded to life. In one word, the mind in man is the mind of the soul." (pp. 13-14.)

We do not think it necessary for us to expose the jumble of ideas contained in this quotation; the looseness of the author's mode of thinking is indicated by his designation of the *actions* of digestion, circulation, respiration, &c. as *properties*. No one can hope to reason successfully on abstract subjects like these, who does not rigidly adhere to the correct use of terms.

The only illustration given of the mode in which the vital agent, residing in the "solar ganglia," (we presume the author means either the semilunar ganglia or the solar plexus,) may be regarded as operating on the body, has reference to muscular movement; by the consideration of which the author states himself to have been led to the views he has adopted. The following is the assumption on which he founds his argument:

"As the mind is not endowed with any innate knowledge of the organized structures, it is very evident that it cannot be the direct cause of any of the muscular movements that are made in the living body. When the mind wills a movement to be made, it may give the stimulus of volition; and the movement is instantly made in obedience to the will. But the movement is not made by the mind, for it can only will, it cannot act or regulate the actions of the muscular fibres. In the involuntary movements it is very clear that the mind cannot be the motor power, and we may rest assured that there is only one principle of action in the animal frame. Even the voluntary actions cannot be made by the mind; for the mind of itself can no more select the proper nerves or move a muscle than it can move a mountain." (p. 7.)

Now on this we have only to remark that, fully admitting our ignorance of the mode in which the will operates upon the nervous system so as to produce muscular motion, we cannot see anything more mysterious about it than in the converse operation, by which a change propagated through a nerve renders the mind conscious of certain sensations; and that neither in the one case nor in the other do we see that any assistance is to be derived from the interposition of a new agent between the mind and the nerve. This vital agent we suppose is considered by its upholder as immaterial; and the difficulty of explaining *its* action on the nerve is, therefore, just as great as in the case of the mind.

The power of the cerebro-spinal system of nerves to excite either voluntary or automatic movements, is entirely derived, according to our author, from its connexion with the sympathetic system; this being, in his estimation, the guide of *all* the actions that take place in the body. Certainly no physiologist, within our knowledge at least, has assigned to it so eminent a function; and until we have some better evidence for this opinion than that at present before us, we must take leave to withhold our assent. We cannot say that we are prepossessed in favour of our author's views, when we find them partly based upon the idea a hundred times refuted, that the ganglionic system of the lower animals is analogous to the sympathetic system of vertebrata; or when we find him stating that "even in the human subject, sensation and the power of motion may continue in the lower extremities, even when the spinal cord has been completely severed from its centre." *Were this true*, he would certainly have some ground for his inference, that "in these cases the muscles in the lower extremities could only be made to obey the will of the mind by some vital power in the ganglionic system." (p. 10.)

The very numerous instances in which experimental physiologists have seriously injured or entirely removed the semilunar ganglia, without the occurrence of any marked results, might have sufficed, we should imagine, to prove the fallacy of the whole theory. In fact we can scarcely suppose that our physiological readers will think it deserving of a serious refutation. The author's total misapprehension of the nature of the reflex function of the spinal cord, which may now take rank as one of the best-established facts in physiology, is shown by the following remark:

"Dr. M. Hall attributes the respiratory movements, when the cerebrum and cerebellum are removed, in mammalia, principally to the reflex action of the pneumogastric nerves. This is evidently an error; for, as the pneumogastric has neither its origin nor termination in, or any connexion with, either the diaphragm or the intercostal muscles, it cannot possibly, under any circumstances, be the direct cause of the respiratory movements." (p. 12, note.)

It is scarcely necessary for us to point out the author's singular mistake in attributing to Dr. M. Hall any such anatomical error; the well-known idea of that physiologist being, that the pneumogastric is the chief *excitor* of the respiratory movements, conveying to the medulla oblongata the stimulus originating in the lungs, which produces a reflex motor impulse along the phrenic and intercostal nerves. It is from this mistake that our author cannot understand why, on Dr. M. Hall's system, the respiratory movements of mammalia should come to a stand, or nearly so, when the cerebrum and cerebellum are removed, and the pneumogastrics and spinal accessory divided. He asks—"Now, if the medulla oblongata or the spinal cord has any inherent power, or if either be the seat of the principle of action, why is it, that under these circumstances neither the one nor both of them can continue the respiratory movements?" No physiologist, unless it be Sir C. Bell, has ever attributed to them any such inherent power; the necessity for a *stimulus* to their action, conveyed by the pneumogastrics from the lungs, having been long recognized. Our author's inference, that the section of the pneumogastric and spinal accessory operates, by cutting off the communication between the sympathetic system and the medulla oblongata, falls, therefore, completely to the ground.

We almost feel that we owe an apology to our readers for detaining them so long upon such a production as this; but we have not commented upon a tenth part of the inaccuracies and unsupported assumptions it contains. To the author, for whom we have great personal respect, we have only to say that it has given us great regret to be obliged to adopt so severe a tone; but we assure him that with us, as with him, truth, not system, is our aim; and that whenever he may give to the world the data on which his views are founded, they shall receive from us an attentive and impartial consideration. In the mean time we would recommend him to devote what leisure he can spare from the "active pursuits of agricultural life," in which he informs us that he is engaged, to making himself acquainted with the actual state of knowledge on the physiology of the nervous system.

